# *Agrobacterium*-Mediated Transformation of Tree Fruit Crops: Methods, Progress, and Challenges

**DOI:** 10.3389/fpls.2019.00226

**Published:** 2019-03-01

**Authors:** Guo-qing Song, Humberto Prieto, Vladimir Orbovic

**Affiliations:** ^1^Department of Horticulture, Plant Biotechnology Resource and Outreach Center, Michigan State University, East Lansing, MI, United States; ^2^Biotechnology Laboratory, La Platina Station, Instituto de Investigaciones Agropecuarias, Santiago de Chile, Chile; ^3^Citrus Research and Education Center, Institute of Food and Agricultural Sciences (IFAS), University of Florida, Lake Alfred, FL, United States

**Keywords:** *Agrobacterium*, genome editing, genetic engineering, genetic transformation, woody fruit and nut crops

## Abstract

Genetic engineering based on *Agrobacterium*-mediated transformation has been a desirable tool to manipulate single or multiple genes of existing genotypes of woody fruit crops, for which conventional breeding is a difficult and lengthy process due to heterozygosity, sexual incompatibility, juvenility, or a lack of natural sources. To date, successful transformation has been reported for many fruit crops. We review the major progress in genetic transformation of these fruit crops made in the past 5 years, emphasizing reproducible transformation protocols as well as the strategies that have been tested in fruit crops. While direct transformation of scion cultivars was mostly used for fruit quality improvement, biotic and abiotic tolerance, and functional gene analysis, transgrafting on genetically modified (GM) rootstocks showed a potential to produce non-GM fruit products. More recently, genome editing technology has demonstrated a potential for gene(s) manipulation of several fruit crops. However, substantial efforts are still needed to produce plants from gene-edited cells, for which tremendous challenge remains in the context of either cell’s recalcitrance to regeneration or inefficient gene-editing due to their polyploidy. We propose that effective transient transformation and efficient regeneration are the key for future utilization of genome editing technologies for improvement of fruit crops.

## Introduction

**FIGURE 1:**
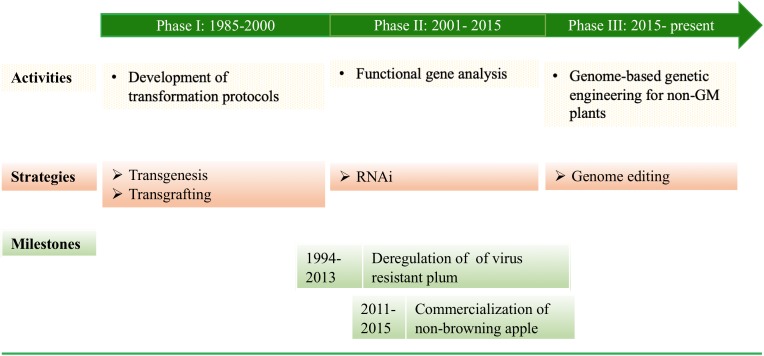
Milestones in genetic transformation of F&N crops.

Fruits and nuts (F&N) provide essential nutrients for human growth and health. There are 494 culinary fruits (excluding fruit vegetables such as watermelons and tomatoes) and 14 nuts listed as of submission in Wikipedia. The increasing consumer awareness regarding health benefits and growing population worldwide are boosting a market for more and higher-quality fruits and nuts. For example, the total global production of fresh fruit increased from 13.6 million metric tons in 1996 to 33.3 million metric tons in 2016 (FAO). On the other hand, many adverse impacts, including emerging diseases (e.g., citrus greening and papaya ringspot virus), abiotic stress (e.g., salt, drought, and extreme temperatures) due to global warming, and natural resource depletion (e.g., land and water) due to the growing population, have threatened fruit yield and quality of F&N crops (Gottwald, 2010; Castillo et al., 2011; Fiore et al., 2018). Thus, the major task for F&N breeders is to develop new cultivars with improved resistance to diseases and abiotic stress, and higher productivity. Conventional breeding for many F&N crops often takes a few to over 10 years due to their long juvenile periods and asexual propagation nature (Janick, 2005). Utilization of biotechnology in breeding is an efficient alternative allowing for the manipulation of gene(s) of interest (GOI) through genetic engineering in shorter period of time, relative to conventional breeding. The efficiency of biotechnology application in breeding has become evident with the successes achieved *via Agrobacterium*-mediated transformation (Figure 1 and Table 1), and carries a great potential with increased availability of sequenced genomes of F&N crops that can be used by technical advances such as gene editing for trait improvement (Table 2).

**Table 1:** Recent successes (2014-present) in *Agrobacterium tumefaciens*-mediated transformation of F&N crops for transformation technology development.

Species	*Agrobacterium* strain	Source explants	Transformation frequency	Reference
*Citrus maximma*	EHA105	*In planta* transformation of 3–4-week old seedlings	3.2–20.4%	Zhang Y.Y. et al., 2017
*Citrus* spp.	EHA105	Microshoots cultured in temporary immersion bioreactor (TIB)	0.25%	Zhang Y.Y. et al., 2017
	EHA105	Internodal stem segments from 30-day old *in vitro* seedlings	3–15-fold increase by expressing of the maize *knotted1* gene	Hu et al., 2016
	EHA101 or EHA105	Mature explants (stems) from bud-grafted mother plant	NA	Orbovic et al., 2015
*Diospyros kaki*	GV3101	Leaves (3–7-week old)	NA	Mo et al., 2015
*Malus micromalus*	EHA105	Cotyledons	11.7%	Dai et al., 2014
*Malus* spp.	GV3101	Young leaves from plantlets	NA	Zhang et al., 2016
*Persea americana*	AGL1	Somatic embryos (globular stage)	NA	Palomo-Rios et al., 2017
*Prunus avium*	EHA105	Leaves from *in vitro* plants	1.2%	Zong et al., 2018
	EHA105	Young leaves	6.4%	Sgamma et al., 2015
*Prunus serotina*	EHA105	Leaf explants	21.7%	Wang and Pijut, 2014
*Prunus domestica*	CBE21	Leaf explants	1.4%	Sidorova et al., 2017
*Pyrus ussuriensis*	EHA105	Buds-leaves from one-year old branches	11.7%	Yang et al., 2017
*Rubus fruticosus*	LBA4404	Cut ends of petioles	5.7–32.4%, results based on PCR analysis of four plants	Sidorova et al., 2017
*Rubus fruticosus*	GV3101	Somatic embryos from whole flower cultures	NA	Dai et al., 2015

**Table 2:** Application of CRISPR-Cas9 gene editing technologies for F&N crops.

Traits	Crop	Gene description	Principle results	Reference
Stable gene transformation-mediated genome editing				
	Apple	Analysis of four separated gRNAs targeting apple phytoene desaturase gene (*PDS*)	31.8% of regenerated transgenic plants for one gRNA induced targeted mutation by *Cas*9	Nishitani et al., 2016
	Grape	Targeting grape L-idonate dehydrogenase gene (*IdnDH*)	Three of six transgenic plants regenerated from 21 stable transgenic cell lines showed targeted mutation	Ren et al., 2016
	Grape	Targeting grape *PDS*	Stable transgenic (T_0_) plants showed targeted mutation in the *VvPDS* gene although chimeric phenotype was observed	Nakajima et al., 2017
	Grape	Targeting grape transcription factor WRKY52	22 targeted mutants were obtained from 72 stable transgenic plants	Wang X.H. et al., 2018
	Kiwifruit	Targeting kiwifruit PDS gene (*AcPDS*)	A demonstration of Cas9-mediated genome editing using paired gRNAs approach for gene fragment deletion in the *AcPDS* gene	Wang Z. et al., 2018
	Citrus	Targeting the promoter region of the susceptibility gene *CsLOB1* and the gene itself for citrus canker resistance	11.5–64.7% mutation rates for five Cas9-constructs (orange), 23.8–89-4% mutation rate for a single Cas-9 construct (grapefruit)	Jia et al., 2017b; Peng et al., 2017
Non-transgene -involved genome editing				
	Apple and grape	Targeting MLO-7, a susceptible gene (S-gene) in order to increase resistance to powdery mildew (PM) in grape cultivar and DIPM-1, DIPM-2 and DIPM-4 in the apple to increase resistance to fire blight disease	PEG-mediated delivery of preassembled Cas9-gRNA reagents resulted targeted mutagenesis in protoplast cells, but no plants with targeted gene editing was obtained	Malnoy et al., 2016


New biotechnological tools revolutionized plant breeding and offered new and effective ways for plant breeders to manipulate traits at the levels of individual gene(s) or gene blocks (Gelvin, 2012; Hiei et al., 2014; Nester, 2014). Except for the widely commercialized virus-resistant papaya produced in 1992 through biolistic-mediated transformation (Fitch et al., 1992), virus-resistant plum (Ravelonandro et al., 1997; Scorza et al., 2001, 2007) and non-browning apples (Waltz, 2015) have been both produced by *Agrobacterium*-mediated transformation. The transgenic plum is on the horizon to be commercially released and the non-browning apples are in the stores. *Agrobacterium*-mediated transformation protocols for stable and transient expression remain a major platform for gene editing technologies in F&N species (Table 1). In this review, we summarize the availability of reliable transformation protocols and discuss recent progress, current constraints, and future perspectives of application of *Agrobacterium*-mediated transformation for the improvement of woody F&N crops.

## History of Genetic Transformation of F&N Crops (Figure 1)

### Phase I (1985–2000)

Development of transformation protocols. After success in *Agrobacterium tumefaciens*-mediated stable transformation of tobacco plants (Horsch et al., 1985), substantial effort was made to develop *A. tumefaciens-*mediated transformation protocols for F&N crops (Kole and Hall, 2008), such as apple (Yao et al., 1995), pear (Mourgues et al., 1996), plum (Mante et al., 1991), cherry rootstock (Gutierrez-Pesce et al., 1998), grapes (Mullins et al., 1990), walnuts (Mcgranahan et al., 1988), kiwifruits (Uematsu et al., 1991), citrus (Cervera et al., 1998), and European chestnuts (Seabra and Pais, 1998).

### Phase II (2001–2015)

RNA interference (RNAi) technologies. The efforts to develop/improve transformation protocols for more F&N crops or cultivars continued and as a result blueberry (Song and Sink, 2004) and sour cherry (Song and Sink, 2006) were transformed. RNAi was used to suppress either virus RNAs or plant endogenous RNAs in plum (Scorza et al., 2004, 2013), cherry (Song et al., 2013), and apple (Saurabh et al., 2014; Waltz, 2015). On the other hand, driven by advances in sequencing, cloning, and RNAi technologies, functional gene analysis became the major focus for the F&N crops, and workable transformation protocols had been developed, including transient expression systems (Scorza et al., 2013). The recombination/excision systems [e.g., Cre*/LoxP* and Flp-*FRT* (flippase recognition target)] have been demonstrated to be effective in producing selectable marker gene (SMG)-free apple (Kost et al., 2015; Krens et al., 2015), apricot (Petri et al., 2012), and citrus (Zou et al., 2013). The most significant progress at this stage include: (1) Deregulation of transgenic plum with plum pox virus (PPV) resistance (Scorza et al., 2007, 2013); and (2) Commercialization of non-browning apples (Waltz, 2015).

### Phase III (2015–Present)

Precision breeding. Gene editing technologies have become powerful tools to precisely manipulate nucleic acids in a plant cell. The very first attempts of these technologies in apple (Nishitani et al., 2016), grape (Ren et al., 2016; Nakajima et al., 2017; Wang X.H. et al., 2018), sweet orange and grapefruit (Jia and Wang, 2014; Zhang F. et al., 2017), and kiwifruit (Wang Z. et al., 2018) have relied on the use of *Agrobacterium* to produce stable transgenic plants expressing either editing reagents or small RNAs inducers. Ideally, transient expression of editing reagents leading to stable editing of a GOI or a regulatory DNA sequence, similar to those demonstrated in annual crops (Svitashev et al., 2016; Liang et al., 2018), will be the next step for F&N plants.

## Transformation Protocols for Woody Fruit and Nut Crops

**FIGURE 2:**
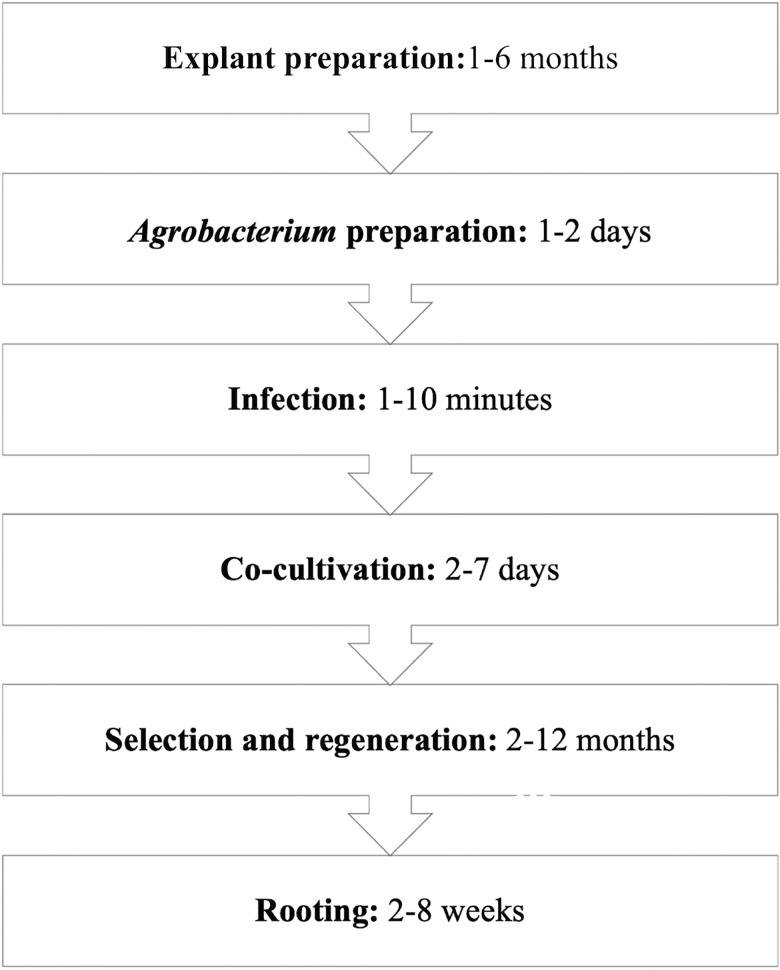
General protocols for *Agrobacterium tumefaciens*-mediated transformation of F&N crops (e.g., blueberry) (Song, 2015).

The current transformation protocols rely on procedures mainly developed between 1990 and 2000. Within the group of F&N species, the majority (over 95%) are still recalcitrant for transformation, and most of the transgenic F&N crops were produced using *A*. *tumefaciens*-mediated transformation (Singh and Sansavini, 1998; Kole and Hall, 2008). The scarce availability of regenerable explant sources and the time required to produce transgenic individuals are the major constraints (Figure 2). For example, only 16 papers on developing or optimizing *Agrobacterium*-mediated transformation of F&N crops have been published since 2014, but regardless of these excellent efforts, little break-through in terms of transformed species and transformation frequency has been made (Table 1).

## Key Limitations for Transformation of F&N Crops

Efficient protocols for transformation rely on effective gene delivery, easy but accurate selection, and prolific regeneration from treated explants (Figure 2).

*Agrobacterium*-mediated gene transfer is a powerful tool for delivery of transgenes although optimal conditions for efficient gene delivery vary for different species, genotypes and strains of *Agrobacterium* (Wang, 2015). Transfer DNA (T-DNA) has been shown to be a consistent carrier for a considerable variety of cargoes ranging from conventional expression cassettes used for GOIs, to the current RNA hairpin inducers (Song et al., 2013) or shuttle vectors for secondary DNA-replicons used in gene editing (Baltes et al., 2014). *A. tumefaciens* is preferable to biolistic guns for stable transformation of F&N crops due mainly to its low cost in operation and the high potential in producing transformations with a low-copy number of the inserted sequence (such as GOI) (Gelvin, 2012). *A. tumefaciens* with ACC deaminase activity has been developed to improve transformation frequency of annual plants through reducing ethylene levels in plants (Nonaka and Ezura, 2014), although it has not been tested in F&N crops. Up to now, gene delivery is not a key limitation for transformation of F&N crops, *A. tumefaciens*-mediated transformation remains the major approach (Tables 1–3).

Within the protocols for transformation of F&N crops, the effective selection is achieved mainly by using a SMG conferring resistance to the plant against either an antibiotic or an herbicide (Miki and McHugh, 2004). Three major SMG for plant transformation include the *neomycin phosphotransferase* II (*npt*II) gene that confers kanamycin resistance, the *hygromycin-B-phosphotransferase* gene (*hph*) conferring hygromycin B resistance, and the *bar* gene conferring resistance to phosphinothricin, a glutamine synthetase inhibitor produced by plants from the herbicide bialophos (Miki and McHugh, 2004). The *npt*II is, to date, the most widely used SMG for the transformation of F&N crops (Kole and Hall, 2008). However, effective selection of transformed cells without assistance of SMGs remains a key limitation for plant transformation.

**FIGURE 3:**
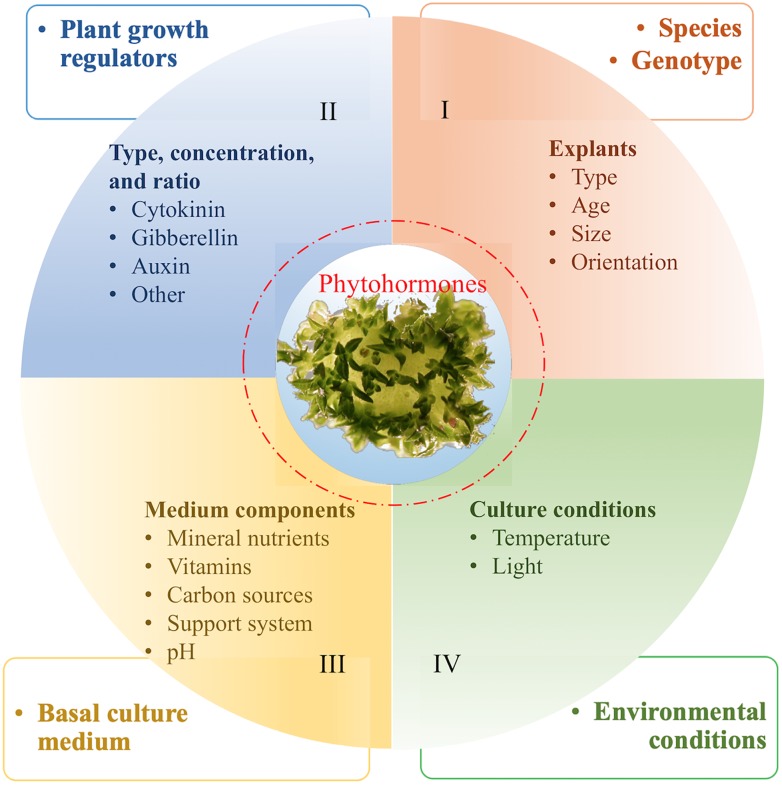
Factors affecting regeneration of plant cells [An example of shoot organogenesis from leaf explants of blueberries (Song and Sink, 2004; Liu et al., 2010)]. We hypothesize that regeneration capability is determined by endogenous phytohormone levels, which are genetically controlled (I) and can be affected by II–IV.

In general, plants with high regeneration capability from non-meristem containing explants are amenable for genetic transformation. Unlike annual crops (e.g., wheat, maize, rice, and soybean), the cultivated F&N crops, for example, blueberries (Song and Hancock, 2012) and cherries (Song, 2014; Zong et al., 2018), are often clonally propagated; to maintain genetic stability, regeneration from explants of clonally propagated tissues is preferable. Lack of available explants sources (e.g., seedlings) makes plant regeneration studies for F&N crops a lengthy process (Figure 3), because in many instances an efficient micropropagation system has to be established for obtaining regenerable explants (e.g., leaves and petioles) prior to regeneration studies (Kole and Hall, 2008). Optimal conditions for both micropropagation and regeneration are determined by many factors (e.g., blueberries) (Liu et al., 2010; Figure 3). In addition, little has been documented on the potential impacts of plant-*Agrobacterium* interaction on plant regeneration. Finally, for some extremely recalcitrant species in the group (e.g., *Prunus persica*) species, regeneration can be achieved mostly on the basis of the use of seed explants (Song, 2014; Petri et al., 2018; Zong et al., 2018). In terms of using transgenes to improve regeneration efficiency, one report demonstrated that constitutive expression of the class I *KNOX* gene of maize increased production of adventitious shoots from leaf explants of plum (Srinivasan et al., 2011). More recently, overexpression of morphogenic regulators, i.e., maize *Baby boom* and maize *Wuschel2*, has been demonstrated to be effective in improving monocot transformation (Artlip et al., 2016); and the potential for use of these genes for F&N crops has been recently demonstrated in citrus (Miki and McHugh, 2004).

## Recent Developments in Genetic Engineering of F&N Crops

Genetic transformation of F&N crops might be necessary when a breeding goal is not easily achievable through traditional breeding approaches (Singh and Sansavini, 1998). Of many approaches developed, gene addition and gene subtraction are two basic strategies for GM trait development (Pena and Seguin, 2001).

**Table 3:** Recent successes (2014-present) in A. tumefaciens-mediated transformation of F&N crops for agronomic trait improvement.

Traits	Crop	Gene description	Principle results^∗^	Reference
Tree architecture	Apple	Apple double-strand RNA-binding protein (MdDRB1) functions in MiRNA processing and maturation	Overexpression of *MdDRB1* promotes adventitious root production and results in columnar-like tree architecture	You et al., 2014
Seed abortion	Grape	The MADS-box gene *VvAGL11* functions in female gametophyte development and fertilization, and seed formation	The mutation of the *VvAGL11* leads to seed abortion	Royo et al., 2018
Non-browning fruit	Apple	Apple polyphenol oxidase (PPO) catalyzes enzymatic browning	Silencing/knock-down the expression of PPO leads to non-browning apple	Waltz, 2015
Yield	Blueberry	*VcSOC1-K*: Keratin-like domain of the MADS-box *SUPPRESSOR* of *OVEREXPRESSION OF CONSTANS 1* (SOC1) gene of blueberry	Overexpression of the MADS-box gene K-domain increases the yield potential of blueberry	Song and Chen, 2018
Flowering time				
	Kiwifruit	Kiwifruit *SHORT VEGETATIVE PHASE3* (*SVP3*) functions in chilling-mediated flowering	Overexpression of the kiwifruit *SVP3* gene affects reproductive development	Wu et al., 2014
	Kiwifruit	Kiwifruit *FT*: a major flowering pathway gene	Early flowering	Moss et al., 2018; Voogd et al., 2017
Abiotic				
	Apple	Apple transcription factor bHLH104 (*MdbHLH104*) regulates transcription	Overexpression of *MdbHLH104* increase apples tolerance to iron deficiency	Zhao et al., 2016
	Apple	Peach C-repeat/DRE binding factor 1 (*PpCBF1*)	Overexpression of *PpCBF1* increases freezing tolerance in transgenic plants	Artlip et al., 2016; Wisniewski et al., 2015
	Apple	Apple dehydration-responsive element binding factor6.2 (*MsDREB6.2*) in stress signaling pathway	Overexpression of *MsDREB6.2* enhances drought tolerance in transgenic apple plants	Liao et al., 2017
	Blueberry	Blueberry *DWARF AND DELAYED FLOWERING 1* (*VcDDF1*)	Overexpression of *VcDDF1* increases freezing tolerance in transgenic blueberry plants	Song and Gao, 2017; Walworth and Song, 2018
	Plum	*Plum stress*-associated protein (PpSAP1)	Overexpression of *PpSAP1* increases water retention under drought stress in transgenic plum plants	Lloret et al., 2017
	Citrus	P35 anti-apoptotic protein	Overexpression of P35 improved cold tolerance	Orbović et al., 2017
Biotic	American chestnut	Wheat oxalate oxidase (*oxo*)	Expression of the *OxO* enhances blight resistance	Newhouse et al., 2014; Newhouse et al., 2018
	Apple	Apple Mildew Locus O19 (*MdMLO19*)	The knock-down of the expression of *MdMLO19* increases apple’s resistance to powdery mildew	Pessina et al., 2016a
	Apple	Fire blight resistance gene of Malus × robusta 5 (*FB_MR5*)	Expression of the *FB_MR5* increases fire blight disease resistance	Kost et al., 2015
	Apple	Chalcone 3-hydroxylase (CH3H) from *Cosmos sulphureus*	Overexpression of the *CH3H* reduces susceptibility to fire blight and scab in transgenic apples	Hutabarat et al., 2016
	Apple	Apple cytosolic malate dehydrogenase gene (*MdcyMDH*)	Overexpression of the *MdcyMDH* enhances tolerance to salt and cold stresses	Wang Q.J. et al., 2016
	Banana	Disease resistance protein (*RGA2*)	Expression of the RCG2 increases resistance to fungus TR4	Dale et al., 2017
	Cherry	*PNRSV-CP*: a partial coat protein gene of Prunus necrotic spot virus	Rootstock-to-scion transfer of transgene-derived small interfering RNAs enables virus resistance in non-transgenic sweet cherry scions	Zhao and Song, 2014
	Citrus	*Mthionine*: a synthetic gene encoding the modified thionin	Overexpression of the *Mthionine* enhances disease resistance to citrus canker and Huanglongbing	Hao et al., 2016
	Citrus	Citrus sinensis lateral organ boundary 1 (*CsLOB1*)	The knock-out of the *CsLOB1* enhances disease resistance to citrus canker	Jia et al., 2017b
	Citrus Grape	*AtNPR1* regulator of systemic acquired resistance Grapevine *E-*(*β*)-caryophyllene synthase (*VvGwECar2*)	Tolerance to HLB disease *VvGwECar2* overexpression results in higher E-(β)-caryophyllene emissions	Robertson et al., 2018 Salvagnin et al., 2016; Salvagnin et al., 2018
	Grape	Grape Mildew Locus O (*VvMLO*s)	The knock-down using RNAi of *VvMLO7* in combination with *VvMLO6* and *VvMLO11* enhances grape’s resistance to powdery mildew	Pessina et al., 2016b
	Grape	A GFLV (grapevine fanleaf virus)-specific nanobody gene *Nb23*	Overexpression of the *Nb23* increases virus resistance to GFLV in grapevine	Hemmer et al., 2018
	Kiwifruit	Synthetic chimeric gene (*SbtCry1Ac*) encodes the insecticidal protein btCrylAc.	Expression of the *SbtCry1Ac* results in insect-resistant plants	Zhang et al., 2015

*^∗^Except non-browning apple that has been commercialized, all the others are proof-of-concept studies.*

### Transgrafting

**FIGURE 4:**
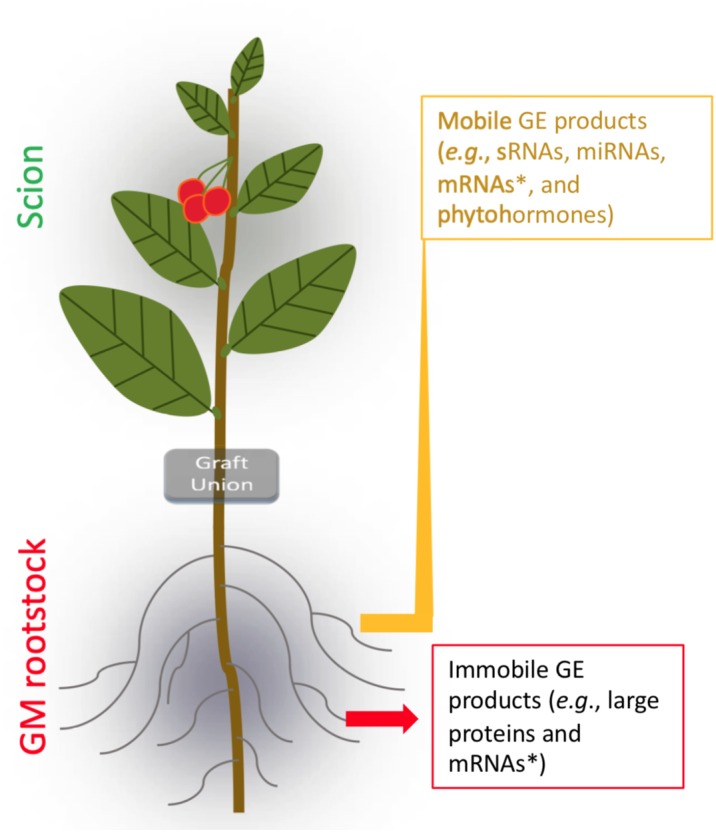
Transgrafting of F&N crops. GE, genetic engineering; SRNAs, small RNAs; miRNAs, micro RNAs. ^∗^Selective targeting movement (Luo et al., 2018).

Grafting by artificially conjoining different vascular systems (i.e., rootstock and scion) is a widely used agricultural practice with over 3000-year history for horticultural crops, especially for F&N crops (Jensen et al., 2012; Nawaz et al., 2016; Chitarra et al., 2017). Traditionally, grafting is used to produce plants for asexual propagation, altered plant vigor and architecture, increased tolerance to biotic/abiotic stresses, precocity, and higher yield. The term transgrafting was introduced when GE rootstocks were used in grafting in 1990 (Haroldsen et al., 2012; Figure 4). To date, long-distance transportation of transgene-derived small interfering RNAs (siRNA) from rootstock to non-transgenic sweet cherry scions have been verified by small RNA sequencing (Zhao and Song, 2014). For large mRNA molecules, qRT-PCR analysis detected short-distance transported mRNAs of the reporter of the red fluorescent protein gene (*DsRED*) from transgenic rootstock to non-transgenic scion of walnut (*Juglans regia*) (Liu et al., 2017); in contrast, for long-distance (>1 m) transportation, the transgene (e.g., the SMG *npt*II gene) was not detected in non-transgenic sweet cherry scions grafted on transgenic rootstock through RT-PCR (Zhao and Song, 2014). More recently, a new study has suggested that cell-to-cell movement of mRNAs is selective (Luo et al., 2018). In fact, interaction of transgenic rootstocks and non-transgenic scions in transgrafted plants through either the mobile transgenic products or immobile transgenic products has been demonstrated to be effective in facilitating changes in non-transgenic scions directly or indirectly (Smolka et al., 2010; Song et al., 2015; Artlip et al., 2016); this is also well-supported by recent studies of grafting in non-transgenic rootstocks and scions (Jensen et al., 2012; Chitarra et al., 2017). Using transgenic apple rootstock expressing the root-inducing *rolB* gene of *Agrobacterium rhizogenes* T-DNA reduced the vegetative growth of nontransgenic scions (Smolka et al., 2010). Thus, transgenic rootstocks have the potential to expand the use of transgenesis for production of non-transgenic F&N crops (Haroldsen et al., 2012).

### FastTrack Breeding

**FIGURE 5:**
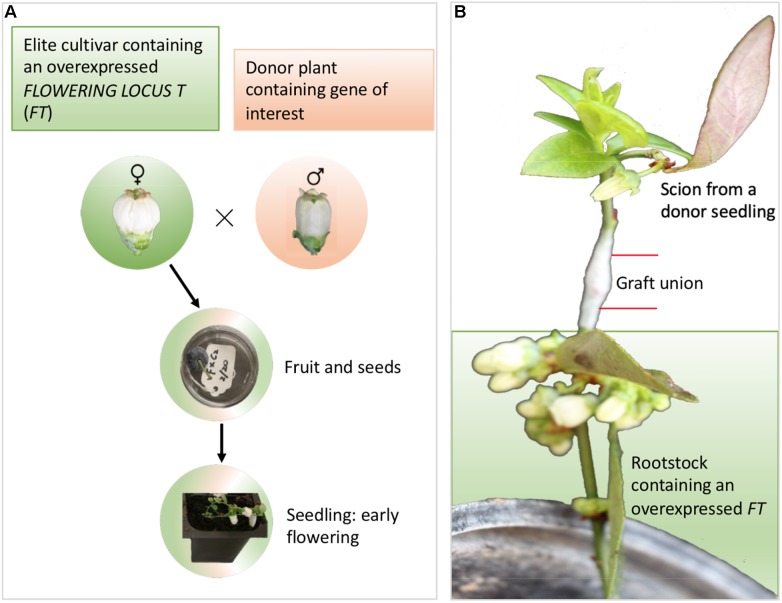
Different approaches for FastTrack breeding – an example for blueberries. **(A)** Stable gene transformation into elite cultivars for overexpression of flower-promoting genes (e.g., *FT*, *SUPPRESSOR OF OVEREXPRESSION OF CONSTANS 1*, *LEAFY*, and *APETALA1*) or repression of flower-repressing genes (e.g., *TERMINAL FLOWER 1).* The early flowering seedlings enable blueberry to skip 2–3 years of juvenile period for each cross. **(B)** Transgrafting on *FT*-overexpressing rootstock promotes early flowering of the scion.

The long juvenile phase exhibited by several F&N crops can severely limit the traditional breeding efforts which are dependent on the ability to make genetic crosses (Janick, 2005). “FastTrack breeding” as demonstrated in many recent publications is done through the manipulation of flowering pathway genes to hasten flowering (Figure 5 and Table 3; Petri et al., 2018). Stable transformation of elite cultivars by either overexpression of flower promoting genes (e.g., *FLOWERING LOCUS T*, *SUPPRESSOR OF OVEREXPRESSION OF CONSTANS 1*, *LEAFY*, and *APETALA1*) or repression of flower repressing genes (e.g., *TERMINAL FLOWER 1*) is an effective approach to enable a fast introduction of genes of interest from wild germplasm through the early flowering seedlings from both crosses and backcrosses (Figure 5A). This approach relies on efficient transformation systems for elite cultivars and low cross-incompatibility between the transformed elite cultivars and donor plants. Transgrafting on transgenic rootstocks can affect flowering in non-transgenic scions (Artlip et al., 2016), suggesting that there is a potential to use a rootstock overexpressing *FT* to promote early flowering of the scions from juvenile seedlings, although this potential has not yet successful been demonstrated for trees (Zhang et al., 2010; Srinivasan et al., 2012; Figure 5B). Alternatively, transient transformation using virus vectors either to enhance expression of flower promoting genes expression or to repress flower repressing genes has showed a potential in promoting flowering of juvenile plants (Yamagishi et al., 2014; Velazquez et al., 2016).

### Selectable Marker Gene-Free Plants and Intragenesis

**FIGURE 6:**
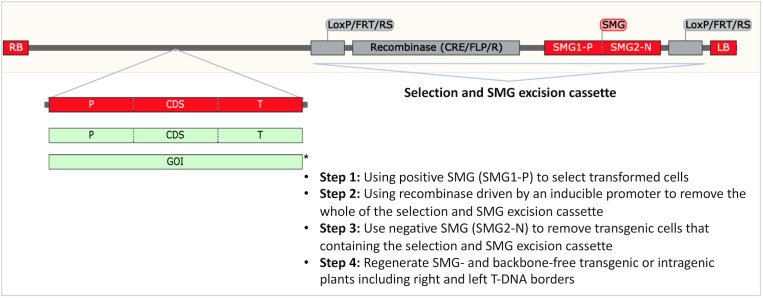
Transgenesis without a SMG and intragenesis. A strategy to obtain SMG free and backbone free transgenic and intragenic F&N crops. The T-DNA region is drawn according to the construct used for intragenic apple (De Paepe et al., 2009; Kost et al., 2015; Collier et al., 2018). This construct allows production of SMG-free T_0_ transformants. RB: right T-DNA border. LB: left T-DNA border. P: promoter. T: terminator. CDS: coding sequence. GOI: gene of interest. Green boxes show plant-derived intragenic/cisgenic components. ^∗^Cisgenesis has not been demonstrated in *A. tumefaciens*-mediated transformation of plants because RB and LB are difficult to replace or remove.

The negative perception of transgenic plants by consumers spurred research to develop either plants modified with plant derived sequences (intragenesis) or transgenic plants from which the SMG was removed (Figure 6). While these strategies have the potential to allay consumer fears, both approaches are currently regulated as transgenic plants worldwide (Holme et al., 2013).

Transgenesis without a SMG or with a plant-derived SMG is preferable to SMG-containing transgenic products by the public (Miki and McHugh, 2004). To date, plant-derived reporters, for example, the genes regulating anthocyanin biosynthesis have been successfully used as alternative to non-plant-derived reporters [i.e., green fluorescent protein (GFP), β-glucuronidase (GUS)] for transformation (Krens et al., 2015; Kandel et al., 2016; Dutt et al., 2018). Co-transformation enables production of SMG-free GM crops through the crosses for the segregation of SMG-removal transformants in next generation, but this is not desirable for asexually propagated F&N crops. The recombination/excision systems [e.g., Cre*/LoxP* and Flp-*FRT* (flippase recognition target)] are effective in generating SMG-free apple (Kost et al., 2015; Krens et al., 2015), apricot (Petri et al., 2012), grape (Dalla Costa et al., 2016), and citrus (Zou et al., 2013).

Intragenesis is desirable for food crops because it relies on the gene pool for conventional breeding. Technically, intragenesis is more challenging than SMG-assisted transgenesis (Figure 6). Previous studies have demonstrated the potential of production of intragenic apples and citrus (An et al., 2013; Kost et al., 2015; Krens et al., 2015).

### Genome Editing

Genome editing technologies provide powerful tools for precise manipulation of targeted genome sequence(s) for crop improvement (Arora and Narula, 2017; Limera et al., 2017). Remarkably, these technologies make it possible to edit or excise a specific gene in a genome without introduction of any extra DNA.

Programmable DNA binding proteins such zinc finger (ZF) and transcription activator-like effector (TALE) emerged as the first generation engineered nucleases to create targeted mutagenesis, which is an alternative to classical protocols for random mutagenesis. These tools have a recognition capability of specific target DNA sequences based on customized arrangements of one (TALE) or three (ZF) nucleotides, and in such way bringing to these places a nuclease (for instance C-terminal domain of *Fok*I) which disrupts DNA adjacent to the recognition zones. Both ZF- and TALE-nucleases, require two effectors (left and right) in order to define the nuclease cutting site (Gaj et al., 2013). Prior to the wide application of these technologies for F&N crops, a more powerful gene editing tool -The clustered regularly interspaced short palindromic repeat (CRISPR)/Cas9 mediated gene editing technology was developed (Jinek et al., 2012). The CRISPR/Cas9 has been a revolutionary molecular tool since its discovery as an adaptive line of defense against viral infection in Archaea (Mojica et al., 2000). This system operates through guide RNAs (gRNAs) that contain specific sequences designed according to their targets in the genome. The Cas nuclease (commonly Cas9), when directed by the gRNA generates a double strand break adjacent to the gRNA’s annealing location allowing for a target-specific mutagenesis. More recently, CRISPR/Cpf1, another CRISPR/Cas system that overcomes some of the CRISPR/Cas9 system limitations, has been found more efficient at DNA editing (Ledford, 2015; Zetsche et al., 2015; Fonfara et al., 2016; Jia et al., 2017a).

Delivery of CRISPR/Cas9 components into the plant cell has been achieved by either transgenic or non-transgenic approaches (Table 2). To date, as proof-of-concept, CRISPR/Cas9 guided DNA editing through stable transformation has been demonstrated in four major fruit crops [i.e., apple (Nishitani et al., 2016), grape (Ren et al., 2016; Nakajima et al., 2017; Wang X.H. et al., 2018), sweet orange and grapefruit (Jia and Wang, 2014; Zhang F. et al., 2017), and kiwifruit (Wang Z. et al., 2018)], suggesting that the CRISPR/Cas9 is suitable for precise gene knockout. The main constraint using stable transformation is that unlike annual crops, from which gene editing associated transgenes can be effectively segregated post editing through traditional crosses, breeding out the transgenes through crosses for F&N crops is undesirable and will eliminate the identity of the clonally propagated variety. Transient transformation without stable integration of the CRISPR/Cas9 components is possible and desirable for gene editing (Chen et al., 2018); whereas technically it is very challenging to identify the targeted mutant cells caused by the CRISPR/Cas9 due to the lack of SMG for transformed plant cells (Table 2). Non-transgene-involved gene editing by using guide gRNA-Cas9/Cpf1 ribonucleoprotein (RNP) complex is ideal for protoplasts of F&N crops and has been demonstrated in apple and grape cells (Malnoy et al., 2016). An alternative approach has been the use of DNA-replicon strategy (Baltes et al., 2014), based on the Bean yellow dwarf virus (BeYDV) genome structure in the absence of proteins required for its infection and mobility (i.e., disarmed virus). This allowed a high copy number in the cell without the insertion of the replicon into the plant genome (Gil-Humanes et al., 2017). Despite these improvements in the genetic engineering area, it is extremely challenging to induce plant regeneration from protoplast cells in F&N species (Table 2). Again, the main challenge for using gene editing technologies to improve the F&N crops remains the lack of efficient regeneration systems.

### Genes and Traits

Regardless of the approach, genetic engineering of the F&N crops aims to modify selected traits through manipulation of targeted gene(s) or gene regulatory sequence(s). The progress made in genetic engineering for fruit crops prior to 2013 has been well-documented (Rai and Shekhawat, 2014).

More recently (2014-present), progress has been made for *A. tumefaciens*-mediated transformation of nine F&N crops using 24 genes for improving seven traits (Table 2). This progress has demonstrated that *A. tumefaciens*-mediated transformation is a powerful tool and remains a major approach for improvement of F&N crops. In this regard, the availability of genome drafts in several F&N species allow for the advance toward improved and safer application of techniques such as RNAi and CRISPR/Cas editing. Interference of target mRNAs using artificial microRNAs, or editing of on-target and effect over off-target *loci* can be assessed by *ex-ante* analyses using dedicated designers which predict these activities in an efficient rate (Doench et al., 2014; Castro et al., 2016). For instance, computing of protospacer adjacent motifs (PAMs) for *Cas*9 over a target genome allows for a first-dimension analysis of the putative cut sites for the nuclease upon gRNA leadership. In that way, Wang et al. (Peng et al., 2017) described the occurrence of more than 35 million PAMs in the grapevine genome and similar developments for other species are becoming available tools (Wang Y. et al., 2016; Pulido-Quetglas et al., 2017).

## Concluding Remarks

Low success rate and recalcitrance of some species to transformation remain as major challenges for adoption of the new breeding techniques to fruit crops. As those challenges are overcome, several new technologies based on *Agrobacterium*-mediated transformation, such as transgrafting, fast-track breeding, intragenesis, and genome editing will be employed more frequently for solving problems facing tree fruit industry worldwide.

## Data Availability

All datasets generated for this study are included in the manuscript and/or the supplementary files.

## Author Contributions

HP wrote the gene editing part. G-qS and VO drafted the rest of the manuscript. All authors read and approved the final manuscript.

## Conflict of Interest Statement

The authors declare that the research was conducted in the absence of any commercial or financial relationships that could be construed as a potential conflict of interest.
